# Highly individual patterns of virus-immune IgG effector responses in humans

**DOI:** 10.1007/s00430-016-0457-y

**Published:** 2016-05-18

**Authors:** Eugenia Corrales-Aguilar, Mirko Trilling, Henrike Reinhard, Valeria Falcone, Albert Zimmermann, Ortwin Adams, Sabine Santibanez, Hartmut Hengel

**Affiliations:** 1 Virology, Research Center for Tropical Diseases (CIET), Faculty of Microbiology, University of Costa Rica, PO Box 11501-2060, San José, Costa Rica; 2 Institute for Virology, University Hospital Essen, University of Duisburg-Essen, Virchowstrasse 179, 45147 Essen, Germany; 3 Institute for Virology, Heinrich-Heine-University Düsseldorf, Moorenstrasse 5, 40225 Düsseldorf, Germany; 4 Institute of Virology, University Medical Center, Albert-Ludwigs-University Freiburg, Hermann-Herder-Strasse 11, 79104 Freiburg, Germany; 5 Division Measles, Mumps, Rubella and Viruses Affecting Immunocompromised Patients, Robert Koch Institute, Nordufer 20, 13353 Berlin, Germany

**Keywords:** Antiviral IgG, FcγRs, ELISA, Neutralization, Measles virus, Human cytomegalovirus

## Abstract

IgG responses are fundamental to adaptive immunity and document immunological memory of previous pathogen encounter. While specific antigen recognition is mediated by the variable F(ab′)_2_ domain of IgG, various effector functions become activated via the constant Fcγ part bridging IgG-opsonized targets to FcγR-expressing immune effector cells. Traditionally, neutralizing IgG is considered the most appropriate correlate of protective humoral immunity to viruses. However, evidence is increasing that antiviral IgG mediates protection to viruses via activation of FcγRs. Using a test system allowing quantitative detection of virus-immune IgG able to activate FcγRs, sera of healthy individuals and vaccinees were assessed with regard to two prototypical human pathogenic viruses: measles and human cytomegalovirus. Marked differences in the capacity of individuals to generate FcγRI-, FcγRII- and FcγRIII-activating responses were noted. Comparison of FcγR-activating IgG with neutralizing and ELISA IgG concentrations did not correlate for HCMV and only very poorly for MV. Since neither neutralizing IgG nor overall IgG responses faithfully predict the activation of FcγRs, only the simultaneous quantification of IgGs activating defined FcγRs will aid to delineate individual “immunograms” of virus IgG immunity. Such new multiparametric assessment of antiviral IgG qualities could be instrumental in defining correlates of protection and disease progression.

**Electronic supplementary material:**

The online version of this article (doi:10.1007/s00430-016-0457-y) contains supplementary material, which is available to authorized users.

## Introduction

The exposure to foreign proteins provokes an antigen-specific adaptive immune response. The humoral arm of adaptive immunity is primarily represented by antibodies (Abs), and among them, IgG molecules play a particularly important role [1]. The high specificity of IgG for its respective antigen allows a retrospective detection of previous pathogen encounters. Therefore, it is common practice to determine antigen specificity of IgG responses for diagnostic purposes or for testing vaccination success. Fab–antigen interactions are usually detected by enzyme-linked immunosorbent assays (ELISA), in which recombinant proteins or protein lysates derived from the respective pathogen are coupled on a solid matrix. These methodologies have undisputable advantages for the rapid identification of pathogen-specific IgG. However, in vivo IgGs must fulfill various immunological effector functions, some of which are Fcγ independent like the recognition of cognate antigen, virus neutralization and opsonization, while others include Fcγ-mediated effects like activation of the complement cascade, induction of phagocytosis and triggering of distinct FcγRs expressed on a large variety of immune cells [1]. Additionally, ELISA-based methodologies have the disadvantage that viral antigens are often not provided in their native conformation and correct membrane topology [2]. The widespread reliance on ELISA-based tests in clinical virology raises the apparent question if and to which extent such data can provide indirect information about the IgG quality in terms of defined effector functions.

Besides ELISA measurements, levels of virus-specific immune IgG are determined by classical in vitro assays like immunofluorescence assays, immunoblots, hemagglutination inhibition and virion neutralization tests, but solely the latter method provides direct information on a defined antiviral effector function that could operate in vivo. The antiviral activity of IgGs observed in vivo has been mainly attributed to virus neutralization where antibodies inhibit virion binding to their entry receptor(s) or prevent fusion between viral and host cell membranes. However, many epitopes exposed on viral or cellular surfaces are not involved in mediating virus entry and fusion and thus do not raise neutralizing Ab responses [3–8]. Irrespective of its neutralizing capabilities, IgG is biologically relevant by eliciting further immune functions (reviewed in Ref. [9]). Adoptive transfer experiments provided a proof of principle for a prominent role of non-neutralizing IgG in controlling primary and recurrent infections, and the absence of detectable virion neutralizing activity within protective antiviral sera further supports the notion that above-mentioned non-neutralizing effector functions are crucial to confine replication of particular viruses, including herpesviruses, MV, poxviruses, LCMV and influenza virus [6, 10–15]. Apart from that, work from the last few years demonstrated the indispensable role of FcγR-mediated effector functions to confer IgG immune protection to various viruses in vivo including murine herpesvirus-68, HIV and influenza [15–20].

FcγRs are exposed on the surface of immune cells. Upon recognition of antigen-bound IgG, FcγRs elicit cell type-specific effector responses such as ADCC, phagocytosis or endocytosis of immune complexes to enhance antigen presentation to T lymphocytes [9, 21, 22]. The family of human FcγRs is composed of FcγRI (CD64), FcγRIIA, FcγRIIB, FcγRIIC (CD32) and FcγRIII (CD16), differing in cellular distribution, affinities for IgG isotypes [23] and effector functions elicited upon activation [24]. FcγRI is found on monocytes and macrophages and binds monomeric IgG with high affinity. FcγRII responds to aggregated IgG and exists in three isoforms either transducing activating (IIA, C) or inhibitory signals (IIB). FcγRIII is expressed on NK cells and macrophages [25] and shows low to medium affinity for immune complexes. While neutralizing IgG prevents virus entry of cells and is therefore thought to be important for establishing “sterilizing” immunity, FcγR-mediated responses become effective upon production and opsonization of viral antigen and thus essential for the control or progression of subsequent steps of virus spread and disease [15, 17, 26–34].

To determine the available fraction of MV- and HCMV-immune IgG being able to activate defined members of the human FcγRs, we applied a set of recently developed reporter cells [35]. Briefly, the assays comprise the co-cultivation of stably transduced FcγR-bearing BW5147 reporter cells with virus-infected target cells displaying native antigens in the presence of IgG. A panel of sera from healthy human individuals was analyzed to relate qualities and quantities of overall MV and HCMV ELISA-reactive IgG to defined antiviral IgG effector functions, e.g., FcγR activation and virion neutralization. Extending beyond the previously described independence of ELISA and PRNT titers [36, 37], only a moderate correlation between global IgG amounts and FcγR activation was found in the case of MV (a serologically monotypic, vaccine-preventable [38] small RNA virus), and no correlation was evident in the case of HCMV (a large herpesvirus encoding an extensive antigenic proteome, for which so far no licensed vaccine exists [39]), indicating that the FcγR-mediated IgG responses cannot be extrapolated from ELISA or plaque reduction neutralization test (PRNT) data. The findings offer new insights into the functional sub-composition of IgG responses against human viruses and highlight the unprecedented effector diversity of antiviral IgG in vitro. Measuring the FcγR-activating capabilities of antiviral IgG increases the prospect to define immune correlates of protection against infections and/or infection-induced disease progression [40].

## Materials and methods

### Cell lines, viruses and infection conditions

Human MRC-5 lung fibroblasts (ATCC CCL-171) and Vero (ATCC CCL-81) cells were maintained in D-MEM (Invitrogen Corp, Life Technologies, Darmstadt, Germany) supplemented with 10 % (v/v) heat-inactivated fetal calf serum (FCS), penicillin (100 U/ml), streptomycin (100 μg/ml) and glutamine (2 mM). All supplements were from Gibco, Life Technologies, Darmstadt, Germany. Mouse BW5147 (ATCC TIB-47™) FcγR-ζ reporter cells [35] were maintained in RPMI 1640 medium containing 10 % (v/v) FCS, penicillin, streptomycin, glutamine and sodium pyruvate (1 mM).

HCMV strain AD169 [41] and the MV strain Edmonston-Enders [42] were used throughout all assays. Infection of cells with HCMV and MV was enhanced by centrifugation at 800 g for 30 min. HCMV and MV infection was performed at 37 and 32 °C, respectively. If not indicated otherwise, cells were infected with 3 PFU/cell to accomplish infection of all cells. Virus-specific CPE was monitored by daily microscopic inspection. Co-cultivation with BW5147:FcγR-ζ reporter cells was started 72 h post-infection and continued for 16 h (see below).

### Human immunoglobulin preparation and human serum samples

A clinically used IVIG preparation, Cytotect^®^ (batch no. A158024, Biotest Pharma GmbH, Dreieich, Germany) containing ELISA-reactive IgG specific for HCMV and MV, was used [43–45]. Cytotect^®^ is manufactured from plasma of healthy volunteer donors (4.500–5.000 donors per batch) from Germany, Austria, Belgium and USA who are selected for high ELISA titers against HCMV. On basis of the very broad selection of donors, Cytotect^®^ was used as a positive polyclonal control for MV and HCMV-IgG in all assays.

Sera of a cohort of 41 donors with unknown immune status against MV and HCMV were used for the determination of individual antiviral IgG response comparisons. These sera were kindly donated by healthy volunteers of unknown MV vaccination status after written consent. Usage of the human sera was approved by the Ethical Board of the Medical Faculty of the Heinrich-Heine-University Düsseldorf (file number. 3054/2008). Donors were randomly selected, and their age varied from 2 months to 90 years (see supplementary Table S1). Another cohort of 18 vaccinees after immunization with MV Triviraten^®^ has been described elsewhere [46]. Briefly, 18 sera obtained from healthy individuals (age between 13 and 15 years) with borderline MV-IgG ELISA reactivity were analyzed concerning FcγR-activating IgG and neutralization capabilities. Sera have been collected during the SCARPOL project [46, 47] with the approval of the Ethical Board of the University of Bern (Switzerland).

### IgG and IgM ELISAs and PRNT assays

Detection of global amounts of virus-specific IgG and IgM was conducted using ELISA tests from Dade Behring (Siemens Healthcare, Erlangen, Germany) [batch no. 36468 (HCMV-IgM), 36294 (MV-IgG), 36364 (MV-IgM)] and from LIAISON DiaSorin (Dietzenbach, Germany) (310.740, batch no. 050045/1 [CMV-IgG]). CMV-IgG titers were detected by LIAISON, and MV-IgG titers were detected by Dade Behring Enzygnost ELISA according to the manufacturer’s instructions. The ELISA test systems are based on inactivated antigen from cells infected with HCMV strain AD169 (www.diasorin.com) or MV strain Edmonston, respectively, to a solid phase support.

PRNT assays for MV and HCMV were performed as described [48–50]. In brief, MV PRNT was performed by preparing serial twofold dilutions of sera or IVIG in minimal essential medium (MEM) alpha medium (Invitrogen, Germany) supplemented with 5  % fetal calf serum (FCS). Mixtures of MV and sera were prepared by adding a serum dilution to an equal volume of an MV suspension containing 40–60 PFU in 100 μl and incubated for 60 min at 37  °C. Aliquots (100 μl) of these mixtures were transferred into cell culture wells with a confluent monolayer of signaling lymphocytic activation molecule (SLAM)-transduced CHO cells and incubated at 37  °C for 60 min. The inoculum was removed, and the monolayers were covered with an overlay containing 0.5  % carboxymethylcellulose and 3  % FCS and incubated for 3 days. The monolayers were stained with crystal violet and fixed with 3.5  % formalin. Plaques were counted visually. For HCMV PRNT, sera and IVIG were twofold diluted in minimal essential medium (MEM) supplemented with 10 % fetal calf serum (FCS). Mixtures of HCMV and sera were prepared by adding a serum dilution to an equal volume of an HCMV suspension containing 40–60 PFU in 100 µl and incubated for 60 min at 37 °C. Aliquots (100 µl) of these mixtures were transferred into a 90 % confluent monolayer of MRC-5 cells, centrifuged for infection enhancement and incubated for 72 h at 37 °C in a 5 % CO_2_ atmosphere. Then, the cells were fixed with pre-cooled methanol, dried and stained for IE HCMV antigens with antibody CCH2 (Dako, Agilent Technologies, Germany). Foci were counted visually. The HCMV- and MV-specific titer denoted for each serum sample and for IVIG in PRNT was calculated as the theoretical dilution resulting in 50 % reduction of the viral plaque or foci number.

### Assessing IgG-dependent activation of the BW:FcγR-ζ reporter cells

The assay used for testing individual IgG-dependent activation of FcγRs is based on co-cultivation of antigen-bearing cells with BW5147 reporter cells stably expressing chimeric FcγR-ζ chain receptors which stimulate mouse IL-2 production in the presence of immune IgG, provided that the opsonizing IgG is able to activate the particular FcγR [35]. For this purpose, IgG-dependent activation of individual BW:FcγR-ζ reporter transfectants was performed by incubating mock and virus-infected cells with serial twofold dilutions of human sera or IVIG in D-MEM 10 % (v/v) FCS for 30 min at 37 °C in an atmosphere of 5 % CO_2_. The range of total IgG concentration used for opsonization varied among viruses (range between 3.5 and 0.0035 mg/ml). To remove non-immune IgG, cells were washed three times with D-MEM containing 10 % (v/v) FCS before co-cultivation with BW:FcγR-ζ reporter cells for 16 h in RPMI 10 % (v/v) FCS medium. If not indicated otherwise, experiments were performed in triplicate and the ratio between reporter (BW:FcγR-ζ cells) and virus-infected target cell was 20:1. After co-cultivation for 16 h at 37 °C in a 5 % CO_2_ atmosphere, supernatants were diluted 1:2 in ELISA sample buffer (PBS with 10 % [v/v] FCS and 0.1 % [v/v] Tween-20) and mIL-2 was measured by ELISA using the capture Ab JES6-1A12 and the biotinylated detection Ab JES6-5H4 (BD Pharmingen™, Erembodegem, Belgium).

To compare the principle reactivity of the different BW:FcγR-ζ reporter cells, cross-linking experiments were performed using grading concentrations of mouse mAbs specific for human CD16-A/B (Clone 3G8, BD Pharmingen, Germany), human CD32 (sc-13527, Santa Cruz Biotechnology, Inc, Heidelberg, Germany) and human CD64 (Clone 10.1 Ancell Corporation, Minnesota, USA) in combination with GAM IgG Fab2 (Sigma-Aldrich, Seelze, Germany) as a secondary reagent (see supplementary Fig. S1).

To determine individual patterns of IgG-mediated FcγR activation, sera were verified regarding the absence of MV- and HCMV-specific IgM by ELISA. Only MV and HCMV-IgM negative sera were further analyzed. Forty-one sera obtained from healthy donors were subjected to HCMV-IgG ELISA (Liaison, DiaSorin) and MV-IgG ELISA (Enzygnost, Dade Behring). Furthermore, PRNT and the BW:FcγR-ζ assays using HCMV strain AD169 or MV strain Edmonston, respectively, were performed. In the BW:FcγR-ζ assays, a serum was regarded as positive, if the concentration of secreted IL-2 significantly exceeded the response of the respective BW:FcγR-ζ reporter cell toward identically infected cells in presence of a serum pool of seronegative donors plus three standard deviations (cutoff) (see supplementary Fig. S2) [35].

### Conceptualization of immunogram and statistical analyses

To allow a direct comparison of the individual assays measuring different antiviral IgG activities and conceptualization of the “immunogram,” the cutoff results of each serum donor in the tests were expressed as percentage of maximal activation (see supplementary Fig. S2). The sample which contained the highest concentration of reactive antibodies was set to 1 (or 100 %). Samples with lower antibody reactivities were assigned accordingly with decreasing percentages until reaching 0 (or 0 %), which were the negative samples. The results of the relative magnitude of responses obtained for IgG ELISA and/or PRNT were used as reference to set the order of sera. This arrangement was kept (irrespective of the actual responses) when the results obtained in other IgG tests are presented. If IgG responses obtained in the reference test (ELISA or PRNT) do predict FcγR-activating IgG titers, the linear correlation should be preserved throughout the other tests when the donors are ordered identically. However, if the results from the reference test are not predictive, the linear correlation should vanish. As an indicator for a potential linear relationship between the respective response and the reference tests (PRNT or ELISA IgG assay, respectively), Pearson’s correlation coefficient (R^2^), which is an indicator for linear relationship between measurements, was calculated.

## Results

### Dissection of MV-immune IgG

To dissect functional IgG response patterns, we decided to begin with a virus producing a restricted array of well-defined antigenic polypeptides after infection and therefore focused on MV. MV represents a serologically monotypic small RNA virus which usually produces a self-limiting acute systemic infection and a long-lasting IgG memory response. Forty-one sera from healthy adult donors were randomly selected and analyzed together with the IVIG preparation Cytotect^®^ by established detection methods, i.e., ELISA for MV-specific IgG (Enzygnost), PRNT, and the newly established test panel for IgG-dependent FcγR activation [35]. All sera included in the study were tested negative for MV-IgM (data not shown), indicating that primary infection events date back. Since the BW:FcγR-ζ reporter cells vary in their maximal IL-2 production upon FcγR engagement, as shown after cross-linking with specific monoclonal antibodies directed against the ectodomain of the respective FcγR (Supplementary Fig. S1 and [35]), results were expressed as relative values compared to the maximal response. The particular serum sample with the strongest reactivity in each of the assay was assigned a value of 1 (or 100 %), and the responses of sera exhibiting less reactivity were ordered accordingly until reaching 0 %, i.e., the value defining a negative result and the absence of this functional type of IgG.

**Fig. 1:**
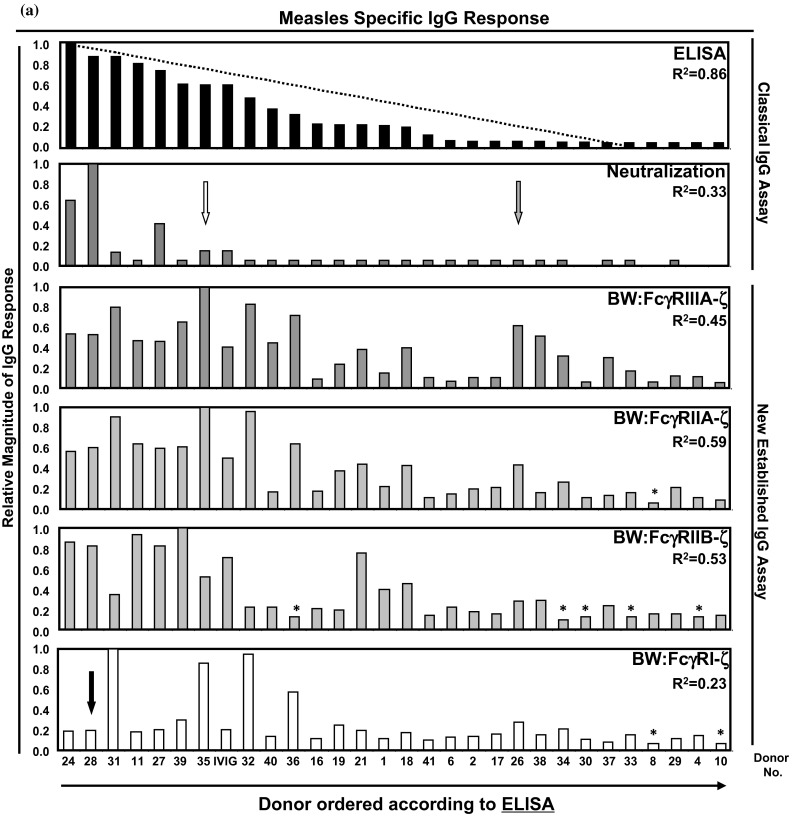

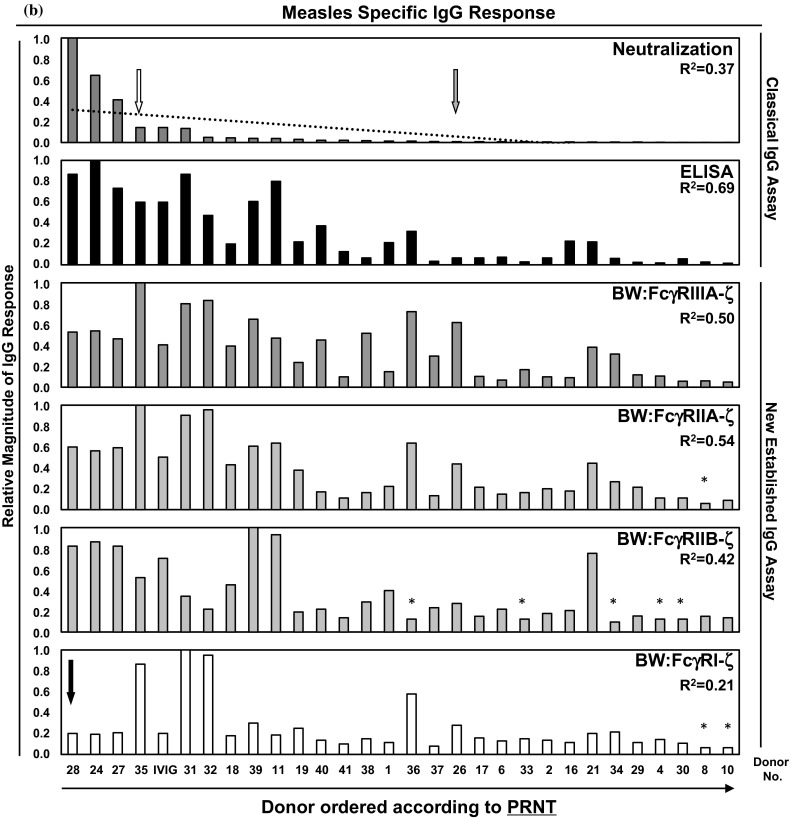
Analysis of measles virus-specific immune IgG reaction patterns of individual human sera. Donor sera were analyzed by the indicated assays for MV-specific IgG responses. The order of the samples was set according to the relative magnitude of the response measured by ELISA (**a**) or PRNT (**b**). For *R* ^2^ values, see figure. Donor no. 28, no. 35 and no. 26 are highlighted by *black*, *white* and *gray arrows*, respectively (see text). *Bars* highlighted by an *asterisk* were below the value defined as positive for that particular assay (see supplementary Table S2). IVIG, Cytotect^®^. *Magnitude of relative IgG response <0.1

Initially, samples were ordered in a decreasing manner according to the reactivity observed in the ELISA IgG assay. In Fig. 1a, the relative values of the sera are depicted in a bar diagram and the order of samples was set depending on their relative response achieved in MV-IgG ELISA as reference test. This order was kept for the other assay formats to assess the predictive value of the reference test for FcγR activation. As observed, the linear correlation seen in the reference test (*R*^2^ = 0.86) vanished when the results of other tests were ordered accordingly (Fig. 1a and Supplementary Table S2 for raw values with standard deviations). This indicates that a donor with a high MV-reactive ELISA titer is not more likely to have high titers of neutralizing or FcγR-activating IgG as well.

The lack of correlation between ELISA and PRNT has been previously described [36, 37] and was explained to result from the fact that MV surface glycoproteins (H, F) contribute less to ELISA reactivity compared to abundant structural internal proteins (N, P) [36]. This argument is also valid in case of FcγR activation, where only surface-exposed antigens can trigger FcγR responses. Therefore, we reordered the samples according to the reactivity observed in the PRNT assay (Fig. 1b). This arrangement resulted in a lower linear correlation value (*R*^2^ = 0.37) due to a rather non-homogenous distribution of measurements caused by few “super-neutralizers” in our cohort distorting the linearity of the statistical evaluation (donors no. 28, 24 and 27, Fig. 1B). FcγRIIIA- and FcγRIIA-activating MV-immune IgG showed a moderate linear correlation (*R*^2^ = 0.50 and 0.54, respectively), whereas FcγRIIB-activating IgG reached only *R*^2^ = 0.42. Likewise, the linear correlation for FcγRI-activating IgG was also low (*R*^2^ = 0.21). On the level of individual donors, the MV-specific IgG profile was quite diverse. For example, donor no. 35 (Fig. 1a, b, white arrow), who had moderate amounts of ELISA-reactive and low neutralizing IgG amounts, exhibited strong FcγR-activating IgG titers. Donor no. 28 (Fig. 1a, b, black arrow) reaching high ELISA reactivity and neutralizing capability exhibited only moderate titers of FcγRIIIA- and FcγRII- but low FcγRI-reactive IgG responses. Donor no. 26 (Fig. 1a, b, gray arrow) showed low ELISA reactivity and very few neutralizing IgGs, but had moderate titers of FcγR reactive IgG. Taken together, the IgG responses measured in the FcγR-ζ activation assays followed the linear trend revealed by the global MV-IgG detected in the PRNT only to a limited extent as indicated by correlation coefficients between 0.21 and 0.54 (Fig. 1a, b). The data indicate that the sub-composition of MV-specific IgG differs considerably among donors with regard to the relative concentration of IgG with neutralizing and FcγR-activating activities, thus resolving individual MV-IgG reaction patterns.

### Dissection of MV-immune IgG in serum samples of borderline ELISA-MV-IgG responses

**Fig. 2:**
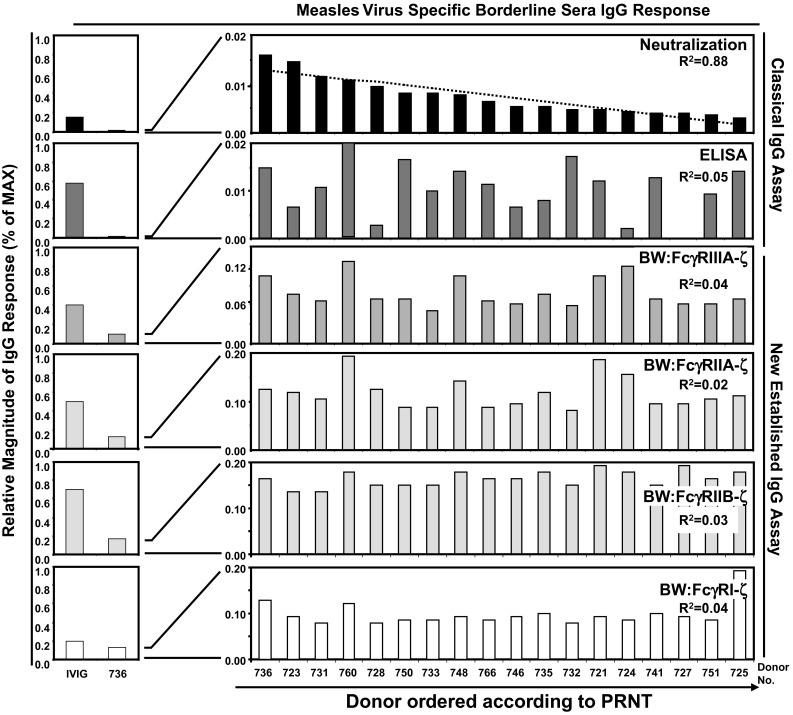
Analysis of measles virus-specific immune IgG reaction patterns of vaccinees with low to undetectable MV-IgG ELISA responses. To compare individual reaction pattern of MV-immune IgG generated in response to a defined live attenuated MV vaccine strain, Triviraten^®^, the serum samples were ordered according to the magnitude of the PRNT response. For *R* ^2^ values see figure. The scale for the relative magnitude of IgG responses (*y* axis) for each assay was set according to the maximal value (MAX) observed within the serum donor panel. This value was compared with the response determined for IVIG (indicated on the *left*). Since the relative magnitude of IgG response for the assays was so low, an amplification of the scale was made. *Left panel* Scale 0–1. *Right panel* Scale 0–0.2 maximal. IVIG, Cytotect^®^

It has been documented that the magnitude of IgG responses to MV differs between individuals with naturally acquired immunity versus those having received vaccination and becomes further modified by booster effects due to subsequent MV exposure [51]. As can be concluded from their broad age distribution (see Supplementary Table S1), donors of the panel investigated for MV-reactive IgG were likely to differ with regard to their MV infection or vaccination history (e.g., infection by different wildtype MV genotypes endemically circulating in Germany which differ with regard to certain neutralizing epitopes [50, 52]), subsequent boosting events and other factors; we next analyzed a well-characterized separate panel of 18 sera obtained from young vaccinees who were selected on the basis to contain neutralizing MV antibodies as determined in sensitive PRNT assays but mounted negative, relatively weakly positive or only borderline ELISA-MV-IgG responses [46]. We hypothesized that these sera containing a higher proportion of MV neutralizing IgG compared to IgG directed against internal MV proteins dominating ELISA responses should be better suited to uncover a potential correlation of NT- and FcγR-activating IgG responses. The PRNT, which is the most sensitive detection method for MV-immune IgG [53, 54], was used as reference test. The ELISA, PRNT and BW:FcγR-ζ reporter cells activation raw values and standard deviations are all listed in the Supplementary Table S3. To compare individual reaction patterns, the sera were ordered according to the percentage achieved in the PRNT (resulting in a linear correlation of *R*^2^ = 0.88 (see Fig. 2)). Again, in a number of sera, the titer of MV-ELISA-reactive IgG did not correlate with the neutralizing IgG (*R*^2^ = 0.05). FcγRIIIA-, FcγRIIA-, FcγRIIB- and FcγRI-activating MV-immune IgG also failed to show a linear correlation with PRNT titers as indicated by *R*^2^ = 0.04, 0.02, 0.30 and 0.04, respectively (see Fig. 2). The data confirmed that the sub-composition of the MV-specific IgG responses among individuals vaccinated with standard doses of a defined attenuated MV vaccine strain is surprisingly heterogeneous, and indicate a lack of clear correlation between FcγR activation and virion neutralizing IgG responses.

### Dissection of effector functions of HCMV-IgG derived from healthy donors

Since we observed discrete albeit only rudimental correlations of functionally defined IgG effector responses against MV, we inferred that in case of antigenically more complex viruses, like herpesviruses, the different effector subtypes of IgG could be even more diverse and less predictable by an assessment of ELISA reactivity. To test this assumption, we measured FcγR-ζ responses of individual sera with unknown HCMV serostatus. Sera from 41 healthy adult donors were analyzed in conjunction with the IVIG preparation Cytotect^®^ by PRNT, ELISA and the novel assays measuring HCMV-IgG-mediated activation of FcγRs. The ELISA, PRNT and BW:FcγR-ζ reporter cells activation raw values and standard deviations are listed in the Supplementary Table S4. All sera were confirmed to be negative for HCMV-IgM (data not shown).

**Fig. 3:**
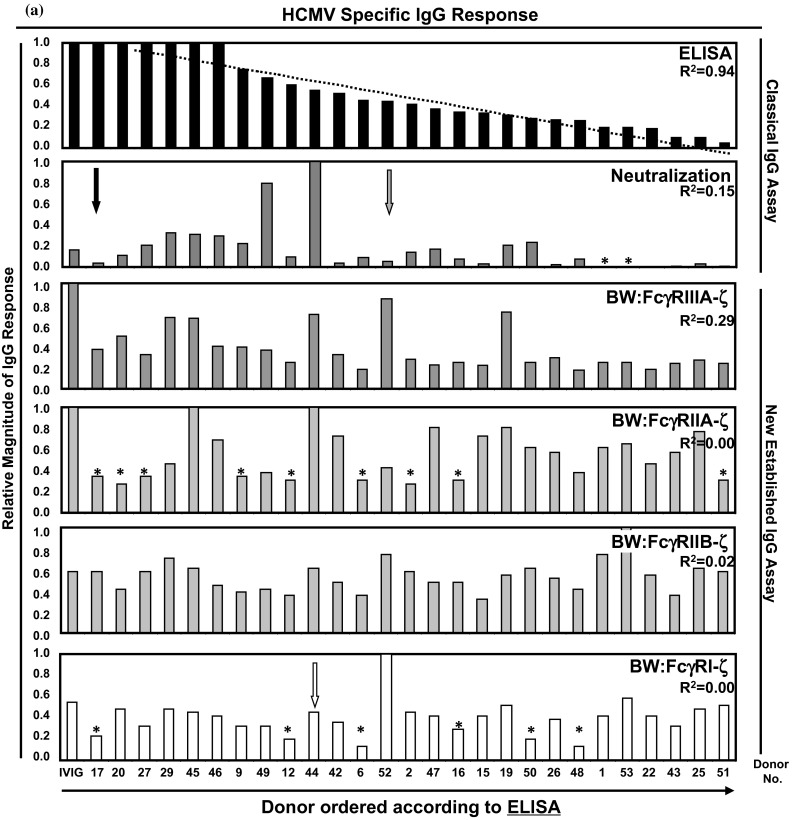

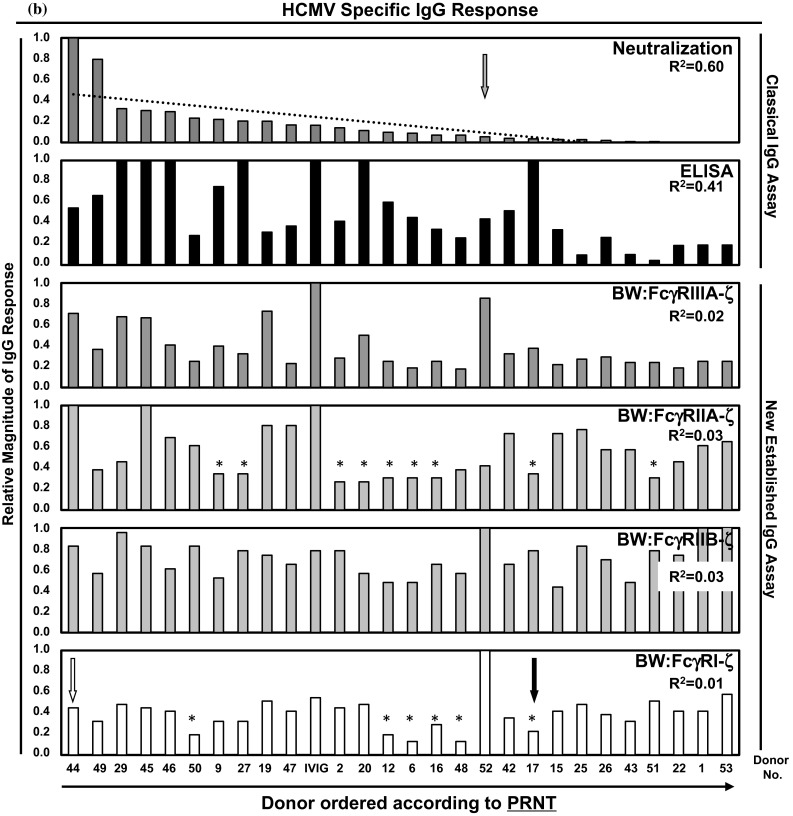
Analysis of HCMV-immune IgG reaction patterns of individual human sera. Donor sera were analyzed by the indicated assays for HCMV-specific IgG responses. The order of the samples was set according to the relative magnitude of the response measured by ELISA (**a**) or PRNT (**b**). For *R* ^2^ values see figure. Donor no. 17, no. 44 and no. 52 are highlighted by *black*, *white* and *gray arrows*, respectively (see text). *Bars* highlighted by an *asterisk* were below the value defined as positive for that particular assay (see supplementary Table S4). IVIG, Cytotect^®^

To unravel reactivity patterns of individual HCMV-IgG donors, the HCMV-IgG ELISA responses were used as reference to order the sera (Fig. 3a). The IVIG pool yielded the maximal response in ELISA, but not in PRNT and only half of the BW:FcγR-ζ assays. Interestingly, individual HCMV-IgG reaction patterns presented a more pronounced diversity as compared to MV-immune IgG. The ordered ELISA data yielded an almost perfect linear trend (*R*^2^ = 0.94). The ELISA test, which was used, is based on the recognition of hypothetically all epitopes of the very large array of antigens expressed by HCMV strain AD 169. PRNT and ELISA exhibited no correlation as indicated by *R*^2^ = 0.15, which can be explained by the fact that antibody responses neutralizing HCMV particles in human fibroblasts are directed to only few HCMV glycoproteins, i.e., the gH/gL/gO complex, gB and gM/gN (reviewed in Ref. [55]). Likewise, very low *R*^2^ values were found when ELISA responses were compared with FcγR activation assessed with opsonized AD169-infected target cells (FcγRIIIA *R*^2^ = 0.29; FcγRIIA *R*^2^ = 0.0001; FcγRIIB *R*^2^ = 0.022; FcγRI *R*^2^ = 0.002), indicating a lack of correlation between these IgG responses.

The antigen display between HCMV particles and the plasma membrane proteome of infected target cells is known to partially overlap [56, 57]. Accordingly, we next ordered the samples pursuant to PRNT values, leading to a moderate linear trend (*R*^2^ = 0.60, see Fig. 3b). When this order of PRNT reactivity was kept constant, while ELISA and FcγR-engaging capacities were plotted, the linear trend diminished or even vanished (Fig. 3b). For ELISA capabilities, the linear correlation dropped to *R*^2^ = 0.41, for FcγRIIIA responses to 0.022, and for FcγRI and FcγRIIA/IIB responses to *R*^2^ = 0.01 and *R*^2^ = 0.03, respectively (Fig. 3b). This documents that neither ELISA nor PRNT titers of anti-CMV IgG are predictive for high FcγR-activating potential. This discrepancy was further substantiated on the single donor level. For example, donor no. 17 (Fig. 3a, b, black arrow) exhibited high levels of HCMV-IgG reactivity in ELISA contrasting with a very low neutralizing capability and low–medium triggering of FcγR responses. Donor no. 44 (Fig. 3a, b, white arrow) exhibited intermediate ELISA reactivity, but reached highest titers of neutralizing and high concentrations of FcγR activation. Donor no. 52 (Fig. 3a, b, gray arrow) exhibited a similar response in ELISA as no. 44, but very low neutralizing IgG, despite high amounts of FcγR-activating IgG. This was most pronounced for FcγRI activation. In conclusion, the responses measured in the FcγR-ζ and virion neutralization assays revealed a surprisingly broad heterogeneity of personal reaction patterns.

### Individual IgG effector profiles are not shared between MV and HCMV

**Fig. 4:**
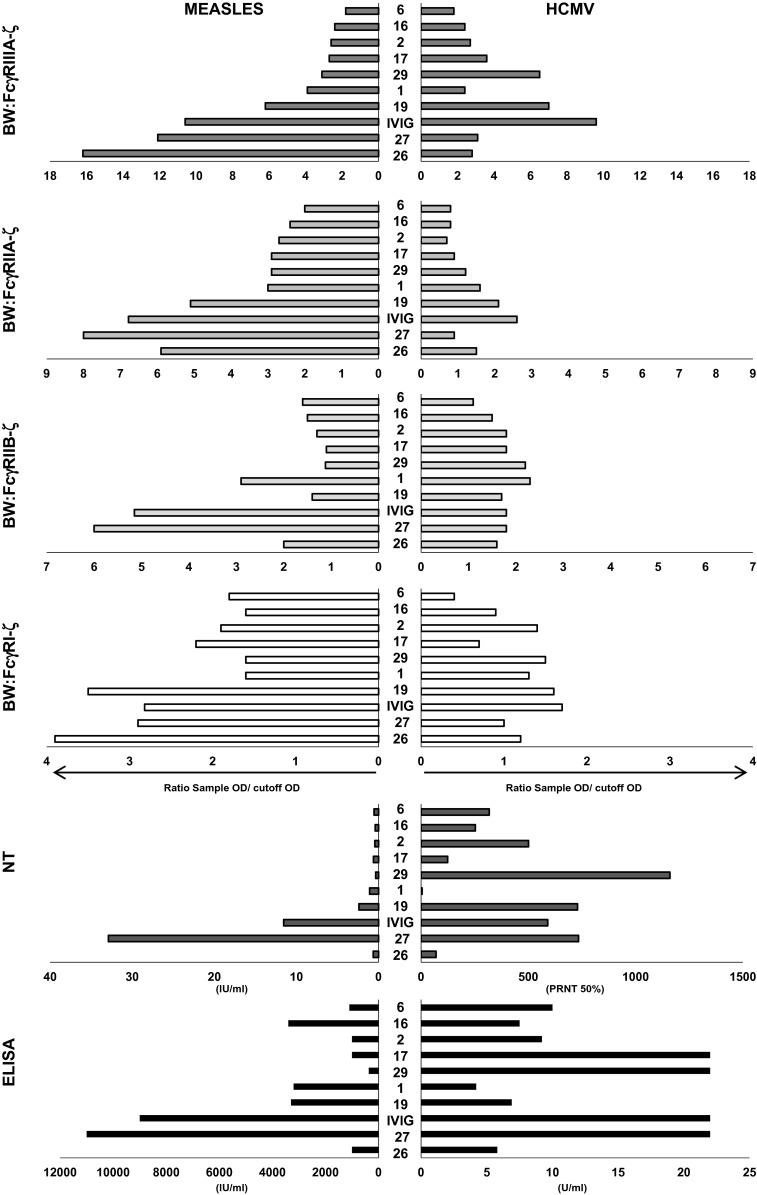
Comparison of HCMV versus MV-immune IgG reaction patterns of individual human sera. Donor sera were analyzed by the indicated assays for HCMV- and MV-specific IgG responses. The order of the samples was set according to the relative magnitude of the response measured by the MV-IgG BW:FcγRIIIA-ζ reporter cell activation assay. IVIG, Cytotect^®^

The observed differences could either be explained by genetic traits (e.g., functionally relevant small nucleotide polymorphisms (SNPs) in immunoglobulin G heavy chain genes or genes involved in IgG N-linked glycosylation) or by different histories of infection and antigen exposure (e.g., different virus strains or boosting events) and subsequent immune reactions. In the first case, one would expect conserved response patterns for different virus infections. To this end, we determined whether the individual IgG effector pattern against one of the tested viruses (e.g., MV) may reflect the IgG effector pattern against the other virus investigated (i.e., HCMV). The comparison was carried out by analyzing nine sera and the IVIG preparation that were found to be reactive in both IgG ELISAs and PRNT against MV and HCMV. The results of the serum samples were expressed as relative values compared to the maximal response, and we ordered the samples according to their reactivity displayed in the MV-IgG BW:FcγRIIIA-ζ reporter cell activation assay (Fig. 4). Evidently, FcγR-activating IgG responses to HCMV were generally lower compared to MV. Furthermore, no correlation between the IgG effector responses against MV and HCMV could be demonstrated. We conclude that the IgG effector pattern observed against one pathogen does not have predictive value for an antigenically unrelated viral pathogen.

## Discussion

Taking advantage of our recently developed comprehensive set of FcγR-ζ chain chimeric reporter cells allowing to detect and quantify virus-immune IgG being able to trigger a specific FcγR (Ref. [34]), we have extensively characterized a panel of human sera from healthy donors. This enabled us to differentiate and to determine the magnitude of IgG effector responses and their potential interrelation with neutralizing antibodies. Two widely different human pathogenic viruses were selected, specifically the paramyxovirus member MV encoding only eight viral proteins, and HCMV, a prototypic β-herpesvirus producing the largest known viral proteome comprising up to 750 translation products [58]. We restricted our analysis of HCMV-IgG responses to the fibroblast-adapted strain AD169 to ensure consistency with the antigens of the commercial ELISA IgG detection system. The analysis of AD169 infection of fibroblasts which is mediated by the glycoproteins gH/gL/gO is likely to imply an underestimate of the overall neutralizing IgG responses present in the sera since HCMV entry of clinical isolates into endothelial and epithelial cells relies primarily on the pentameric complex (gH/gL/UL128/UL130/UL131) which is targeted by a majority of the neutralizing IgG [59], but could also be present on infected cells. The analysis of HCMV strains with intact pentamers in our set of assay systems is therefore an obvious task in the future. Despite the elimination of this additional level of antigenic variability due to the pentamer-deficient AD169 strain used here in both the PRNT and FcγR activation assays, surprisingly, no correlation between FcγR-activating and neutralizing IgG responses was noted. This was also not the case in a quite homogenous cohort of teenage vaccinees upon MV vaccine uptake [46] exhibiting only low or no detectable ELISA-MV-IgG responses. The latter are thought to be dominated by antibodies recognizing internal MV proteins [60], a fact that could possibly impede the analysis of IgG effector responses to surface MV glycoprotein antigens H and F which are targeted by neutralizing [61] as well as FcγR-activating IgG.

### Why do neutralizing IgG responses hardly correlate with FcγR activation by opsonizing IgG?

Several explanations are possible for this unexpected finding. The epitopes recognized by neutralizing versus FcγR-activating IgG could differ in several aspects, e.g., (1) their number on viral entry proteins, (2) their localization and (3) the number of IgG ligands that are required to mediate one particular response. While defined biochemical features of IgG molecules like their N-linked glycan linkage at Asn297 and the IgG subclass assignment are known to be highly crucial for FcγR interaction [9, 23], neutralization of virions is determined by the physical interference of bound IgG with the concerted structural changes of host and viral proteins mediating the viral entry process. Viral fusion proteins are central in this sequence of events. On the intact virion, the fusion proteins are in a high-energy metastable pre-fusion conformation, while during infection the protein undergoes numerous transitions resulting in a more stable lower-energy post-fusion conformation of the protein. As deduced from available crystal structures [62], the extensive structural rearrangement of involved fusion proteins is associated with important alterations in the formation and accessibility of epitopes present in the pre- versus post-fusion conformation. Therefore, the IgG clonotypes recognizing the pre-fusion protein displayed on infectious virions and those opsonizing the post-fusion protein exhibited on the surface of infected cells will differ. The contribution of different IgG clonotypes to neutralizing versus FcγR-activating responses may contribute to the discrepant reaction patterns observed. In many virus infections, the emergence of IgGs that modify the efficacy of neutralizing IgGs as “interfering antibodies” or “enhancing antibodies” has been documented [63]. It is obvious that those IgGs could contribute to activate FcγRs and thus act on the contrary to neutralizing IgG.

**Fig. 5:**
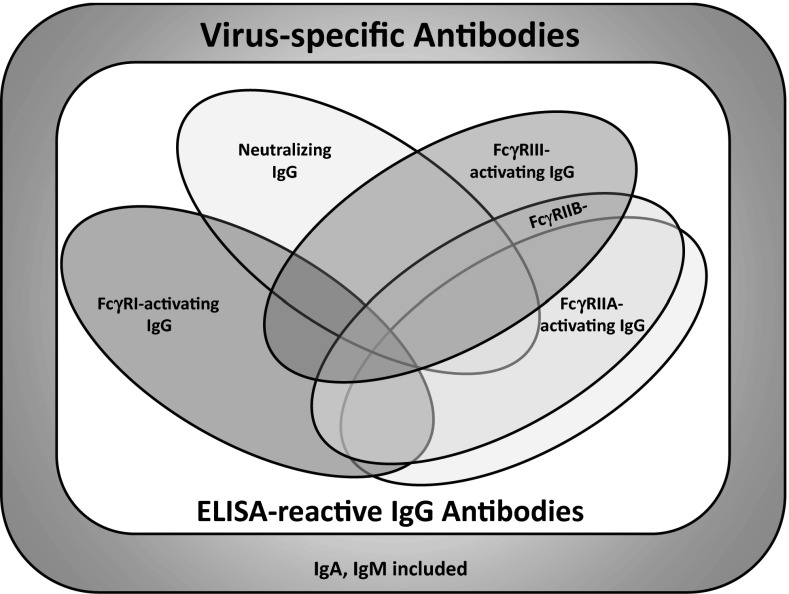
Sub-composition of the virus-specific antibody response—conceptualization of an “immunogram.” As part of the total amount of serum antibodies recognizing a given virus, the pool of virus-immune IgG is detectable by ELISA depending on the array of viral antigens represented in the test and the biophysical binding properties of immune IgGs. Within the ELISA-reactive IgG fraction, some virus-immune IgG clonotypes possess distinct functional properties, i.e., virion neutralization or activation of specific FcγRs (FcγRIIIA/CD16 and/or FcγRIIA/CD32A and/or FcγRIIB/CD32B and/or FcγRI/CD64) upon recognition of viral epitopes. Some IgGs may exhibit overlapping functional features. In addition to IgG, some IgA and IgM antibodies recognizing virion surface epitopes can be neutralizing

The individual pattern observed for the FcγRI-, FcγRIIA-, FcγRIIB- and FcγRIIIA-mediated responses was less diverse as compared to neutralizing or ELISA IgG responses, but still differed substantially. Since IgG subclasses and Asn297 glycans have crucial influence on the relative capacity of IgG molecules to trigger FcγRs, the analysis of the subclass composition and Asn297 glycan structures of virus-specific IgGs should allow more insight how individual “immunograms” (Fig. 5) are constructed.

### Intramolecular IgG interactions and viral inhibitors influence FcγR activation

Despite continuous exposure of FcγR-bearing immune cells to high titers of serum IgG, these cells become only activated upon pathogen encounter. This indicates that F(ab′)_2_-dependent recognition of the cognate antigens must instruct molecular changes (e.g., either by local clustering or conformation changes within the IgG molecule) which are sensed by FcγRs. Consistently, it has been shown that the binding of staphylococcal protein A and streptococcal protein G to C_H_1 and C_H_2–C_H_3 domains of IgG1 is affected by recognition of the specific antigen [64], challenging the traditional view of the F(ab′)_2_ and Fc domains as structurally and functionally independent modules (reviewed in [65]). It is thus tempting to speculate that differences in the nature of the epitope–paratope interaction (in terms of affinity, avidity and availability) might result in differential FcγR activation. As documented before [37, 54, 66], overall MV-specific IgG responses as determined by standard whole-cell ELISA or proteome microarrays [60] largely failed to predict neutralizing IgG effector responses. Here we document that this is also found true for FcγRI/II/III-activating MV-IgG. In this context, it is of interest that Kim et al. [67] found that inhibition of MV vaccination by maternal IgG seems not to be caused by masking of neutralizing epitopes as previously thought. Rather, the inhibition of B cell responses by MV-specific IgG occurs via binding to the inhibitory FcγRIIB, emphasizing the need to discriminate between neutralizing and FcγRIIB-mediated IgG effector functions. Despite the great variability of FcγR-mediated responses observed between serum donors, a consistent discrepancy was noticed between HCMV- and MV-specific responses. As a clear trend seen within MV and HCMV double seropositive donors, MV-infected cells were considerably more potent to activate FcγRs when compared with HCMV-infected cells (Fig. 4). This effect can be attributed to the expression of HCMV-encoded antagonists of FcγR activation, e.g., *RL11*/gp34 and *UL119*-*118*/gp68 [68–70]. The presence of these counteracting immune-evasive molecules targeting ADCC responses highlights the antiviral potency of FcγR-dependent IgG responses which put HCMV under constant immune selection pressure [71].

### Striving for a refined diagnosis system of antiviral IgG

Virus-specific IgG constitutes a pillar of immunity, and its administration to non-immune individuals can alleviate disease or even prevent virus transmission [44, 72, 73]. However, ELISA-based measurements of IgG titers have often failed to predict the clinical outcome of particular viral infections in humans and to serve as a reliable surrogate marker of immune protection [74–76]. We surmise that this could be based on the fact that functionally diverse but partially overlapping sub-fractions of IgG molecules to a given virus exist (see Fig. 5, “immunogram”) which may have unequally distributed impact on virus immune control. Accordingly, the measurement of global amounts of IgG physically bound to viral antigens as in ELISA test formats constitutes only a vague attempt to assess a distinct correlate of antiviral immunity. A steadily growing number of studies support the notion that FcγR-dependent immunity is crucially involved in antiviral control [6, 10–15] and vaccine responses [77] but may be also required for successful IgG treatment of tumors [78–80], as well as mediating anti-inflammatory effects of intravenous IgG [81, 82]. Hypothesizing that FcγR-activating IgG responses execute a relevant yet still ill-defined immune effector function, we set out to investigate (1) the proportion of such antibodies among the total amount of polyclonal IgG directed against a given virus and (2) the quantitative ratios between the definable IgG effector functions within a cohort of healthy individuals. Our findings reveal a large variety of individual effector profiles for virus-immune IgG rather than homogeneous reaction pattern against one particular virus or consistent effector profiles across different pathogens within one individual (see Figs. 4, 5).

### Future validation of FcγR-activating IgG responses as a correlate of immune protection

Animal models are instrumental to better define distinct IgG effector functions as mechanistic correlates of antiviral immunity and protection and thus generate hypotheses for clinical situations in humans including the more precise assessment of successful vaccine responses [3, 5, 6, 15–17, 77]. Investigating different inbred mouse strains infected with mouse cytomegalovirus (MCMV), we observed marked interstrain-dependent differences (“immunograms”) of FcγR-dependent IgG immunity resembling the situation in humans (G. Androsiac, H. Hengel, unpublished observation). Assessment of the individual FcγR-activating profiles of anti-influenza virus-specific mAbs sharing identical antigen specificity but differing in their IgG subclass assignment correlated surprisingly well with their varying protection capacity in lethally influenza virus-infected mice lacking specific FcγRs (S. Van den Hoecke, K. Ehrhardt, H. Hengel, X. Saelens, unpublished observation). In a next step, further animal studies should disclose whether this predictive accuracy of FcγR-activating IgG responses determined by our assays can be verified in reference to polyclonal IgG responses and further viral pathogens.

## Electronic supplementary material

Below is the link to the electronic supplementary material.

Fig S1 **Cross-linking experiments with mAb directed against the ectodomain of Fc**γ**Rs prove heterogeneity in activation**. Goat antimouse IgG F(ab)_2_ was coated onto 96-well cell culture plates (2 µg/ml). After blocking and washing, mouse mAbs specific for human CD16-A/B, human CD32 and human CD64 were added. As a negative control, GAM F(ab)_2_ was used. After removal of unbound antibodies, 2 × 10^5^ BW:FcγR-ζ transfectants were added per well. mIL-2 secretion (OD 450 nm) was determined after 16 h. n = 3 (PPTX 53 kb)

Fig S2 **General approach how individual IgG responses were compared and ordered**. In the example shown, BW:FcγRIIIAζ responses (upper panel) were measured as amount of produced IL-2 upon co-cultivation of reporter cells with MV-infected Vero cells (3 PFU/cell, infected for 72 h) opsonized with serum from donors #35, #33, #31 and #3 at the indicated dilutions (step 1). A serum was regarded as positive if the concentration of secreted IL-2 exceeded the response of the respective BW:FcγR-ζ reporter cell toward identically infected cells in the presence of a serum pool of seronegative donors plus three standard deviations (cutoff, as indicated). In this example, the cutoff value was 0.085. Ordering of positive sera #35, #33 and #31 was based on fold of cutoff values determined at 1:20 dilution (step 2). Serum # 35 reached a value of 26.1-fold of the cutoff. Step 3: The sample which yielded the highest response was set to 1 (or 100 %). In this example, serum #35 was ranked as number 1, and samples with lower reactivities were expressed with relative values, e.g., sample #33 has a rank of 4.4/26.1 = 0.17. Step 4: The order of sera obtained in the BW:FcγRIIIA-ζ analysis was kept when compared with MV-ELISA results (lower panel) (PPTX 1544 kb)

Supplementary material 3 (PDF 9 kb)

Supplementary material 4 (PDF 311 kb)

Supplementary material 5 (PDF 241 kb)

Supplementary material 6 (PDF 312 kb)
