# Supplementary material for: Highly individual patterns of virus-immune IgG effector responses in humans
Source: Med Microbiol Immunol. 2016 May 18;205(5):409–24. doi: 10.1007/s00430-016-0457-y (PMC5003914; doi:10.1007/s00430-016-0457-y)
Supplement: Supplementary file 1 — Cross-linking experiments with mAb directed against the ectodomain of FcγRs prove heterogeneity in activation. Goat antimouse IgG F(ab)2 was coated onto 96-well cell culture plates (2 µg/ml). After blocking and washing, mouse mAbs specific for human CD16-A/B, human CD32 and human CD64 were added. As a negative control, GAM F(ab)2 was used. After removal of unbound antibodies, 2 × 10^5^ BW:FcγR-ζ transfectants were added per well. mIL-2 secretion (OD 450 nm) was determined after 16 h. n = 3 (PPTX 53 kb) [file 430_2016_457_MOESM1_ESM.pptx]

## Slide 1
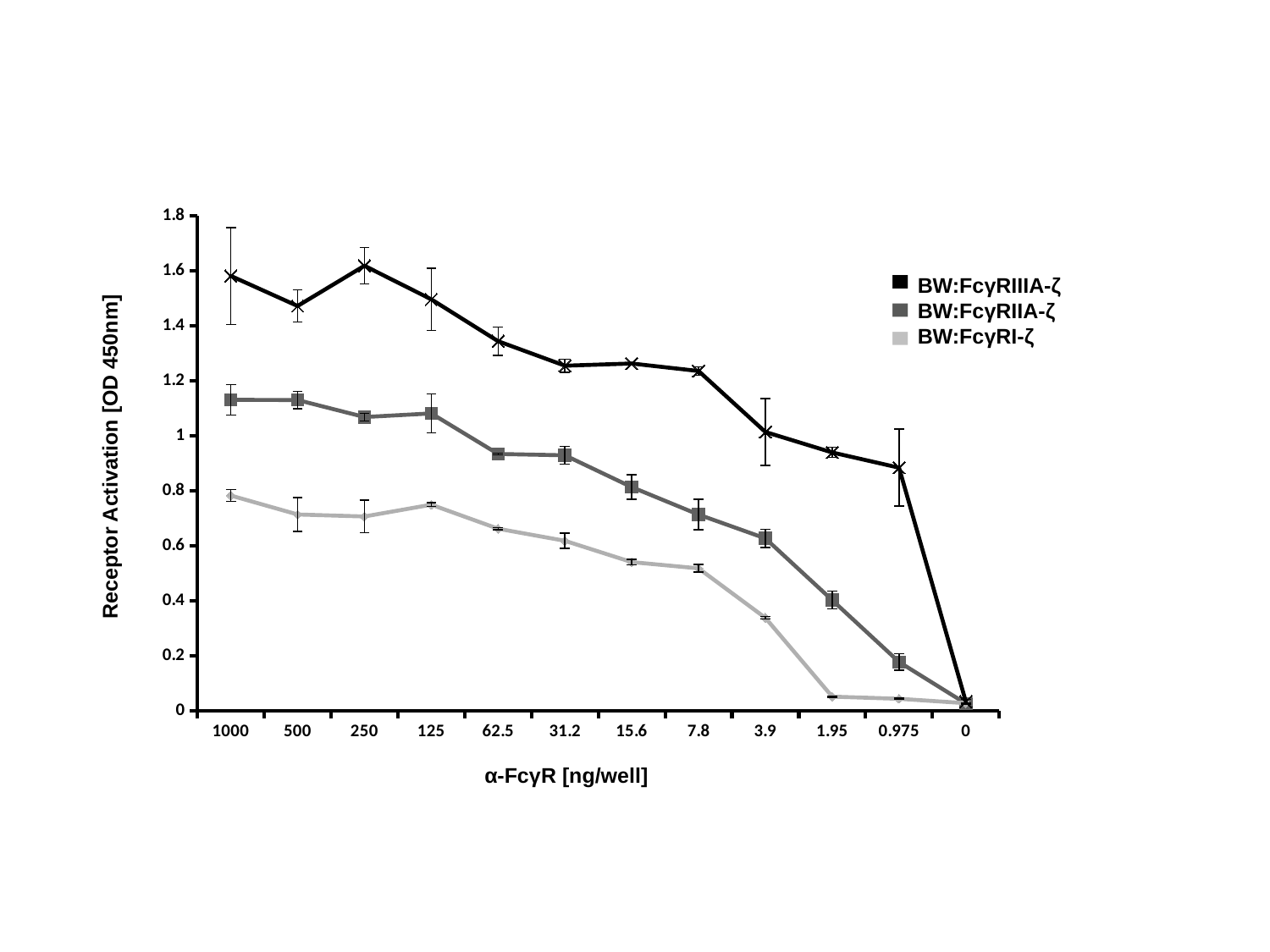

### Chart
| Category | | | |
|---|---|---|---|
| 1000 | 1.1304500102996826 | 0.7821000218391418 | 1.5811499953269958 |
| 500 | 1.1297500133514404 | 0.7132500112056732 | 1.471750020980835 |
| 250 | 1.0678499937057495 | 0.7058999836444855 | 1.618149995803833 |
| 125 | 1.0809999704360962 | 0.748850017786026 | 1.4958999752998352 |
| 62.5 | 0.9334499835968018 | 0.6615000069141388 | 1.343649983406067 |
| 31.2 | 0.9285500049591064 | 0.6175000071525574 | 1.2545999884605408 |
| 15.6 | 0.8127500116825104 | 0.539900004863739 | 1.2626000046730042 |
| 7.8 | 0.7129000127315521 | 0.517300009727478 | 1.2353500127792358 |
| 3.9 | 0.6263500154018402 | 0.3373500108718872 | 1.0134999752044678 |
| 1.95 | 0.4016999900341034 | 0.05065000057220459 | 0.9390499889850616 |
| 0.975 | 0.17660000175237656 | 0.04274999909102917 | 0.8835499882698059 |
| 0 | 0.025500000454485416 | 0.026750000193715096 | 0.032349999994039536 |
BW:FcγRIIIA-ζ
BW:FcγRIIA-ζ
BW:FcγRI-ζ
Receptor Activation [OD 450nm]
 α-FcγR [ng/well]
