# Supplementary material for: Highly individual patterns of virus-immune IgG effector responses in humans
Source: Med Microbiol Immunol. 2016 May 18;205(5):409–24. doi: 10.1007/s00430-016-0457-y (PMC5003914; doi:10.1007/s00430-016-0457-y)
Supplement: Supplementary file 2 — General approach how individual IgG responses were compared and ordered. In the example shown, BW:FcγRIIIAζ responses (upper panel) were measured as amount of produced IL-2 upon co-cultivation of reporter cells with MV-infected Vero cells (3 PFU/cell, infected for 72 h) opsonized with serum from donors #35, #33, #31 and #3 at the indicated dilutions (step 1). A serum was regarded as positive if the concentration of secreted IL-2 exceeded the response of the respective BW:FcγR-ζ reporter cell toward identically infected cells in the presence of a serum pool of seronegative donors plus three standard deviations (cutoff, as indicated). In this example, the cutoff value was 0.085. Ordering of positive sera #35, #33 and #31 was based on fold of cutoff values determined at 1:20 dilution (step 2). Serum # 35 reached a value of 26.1-fold of the cutoff. Step 3: The sample which yielded the highest response was set to 1 (or 100 %). In this example, serum #35 was ranked as number 1, and samples with lower reactivities were expressed with relative values, e.g., sample #33 has a rank of 4.4/26.1 = 0.17. Step 4: The order of sera obtained in the BW:FcγRIIIA-ζ analysis was kept when compared with MV-ELISA results (lower panel) (PPTX 1544 kb) [file 430_2016_457_MOESM2_ESM.pptx]

## Slide 1
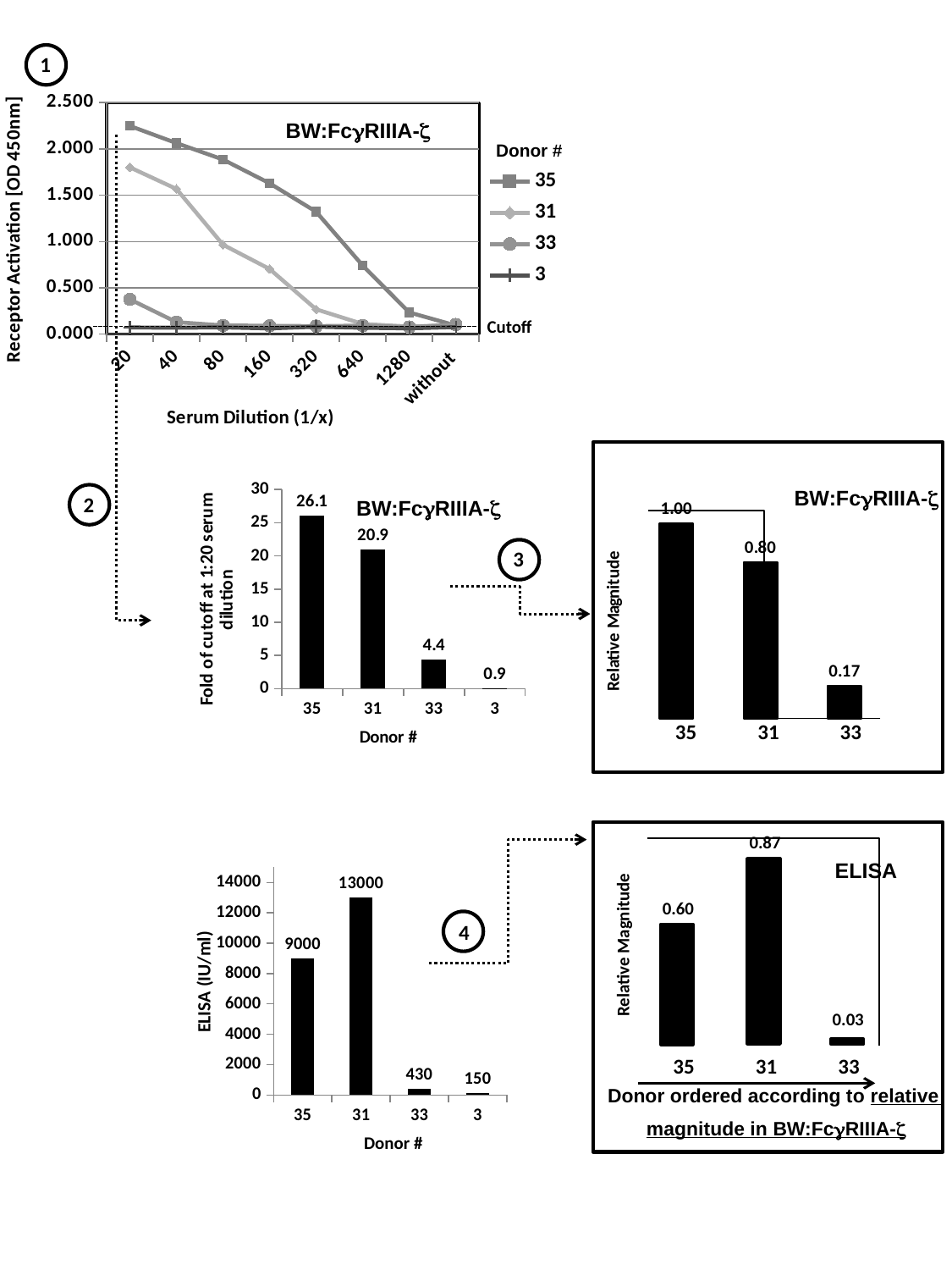

1
### Chart
| Category | 35 | 31 | 33 | 3 |
|---|---|---|---|---|
| 20 | 2.247 | 1.8 | 0.3755 | 0.0725 |
| 40 | 2.0615 | 1.5670000000000002 | 0.1295 | 0.0695 |
| 80 | 1.8845 | 0.965 | 0.0945 | 0.073 |
| 160 | 1.6280000000000001 | 0.7035 | 0.0905 | 0.062 |
| 320 | 1.3215 | 0.26849999999999996 | 0.0865 | 0.08 |
| 640 | 0.7415 | 0.1105 | 0.0925 | 0.069 |
| 1280 | 0.2345 | 0.0825 | 0.077 | 0.0625 |
| without | 0.0935 | 0.095 | 0.1045 | 0.0765 | BW:FcgRIIIA-z
Donor #
Cutoff
### Chart
| Category | |
|---|---|
| 35 | 26.1 |
| 31 | 20.9 |
| 33 | 4.4 |
| 3 | 0.09 |
### Chart
| Category | |
|---|---|
| 35 | 1.0 |
| 31 | 0.8001531393568146 |
| 33 | 0.16845329249617153 |2
 BW:FcgRIIIA-z
 BW:FcgRIIIA-z
3
35
31
33
0.87
0.60
0.03
35
31
33
### Chart
| Category | |
|---|---|
| 35 | 9000.0 |
| 31 | 13000.0 |
| 33 | 430.0 |
| 3 | 150.0 | ELISA
4
Relative Magnitude
ELISA (IU/ml)
Donor ordered according to relative
magnitude in BW:FcgRIIIA-z
Donor #
