# Supplementary material for: Highly individual patterns of virus-immune IgG effector responses in humans
Source: Med Microbiol Immunol. 2016 May 18;205(5):409–24. doi: 10.1007/s00430-016-0457-y (PMC5003914; doi:10.1007/s00430-016-0457-y)
Supplement: Supplementary file 3 — Supplementary material 3 (PDF 9 kb) [file 430_2016_457_MOESM3_ESM.pdf]

Table S1: Sex and age distribution of donors

		Sex		Age	
		**Female**	**Male**	**Median**	**SD**
**Measles**	**Total**	24	17	27.1	16.2
	**Positive**	18	11	28.9	17.0
	**Negative**	6	6	22.8	14.0
**HCMV**	**Total**	25	16	29.1	18.8
	**Positive**	16	11	32.2	19.7
	**Negative**	9	5	23.1	15.7

Table S1: Female/male distribution of the 41 serum donors. Mean age.
