# Supplementary material for: Highly individual patterns of virus-immune IgG effector responses in humans
Source: Med Microbiol Immunol. 2016 May 18;205(5):409–24. doi: 10.1007/s00430-016-0457-y (PMC5003914; doi:10.1007/s00430-016-0457-y)
Supplement: Supplementary file 4 — Supplementary material 4 (PDF 311 kb) [file 430_2016_457_MOESM4_ESM.pdf]

Table S2: Raw data for MV immune IgG reaction patterns of individual human sera.

						**FcγRIIIA-ζ activation**			**FcγRIIA-ζ activation**			**FcγRIIB-ζ activation**			**FcγRI-ζ activation**			
**Donor no.**	**Sex**	**Age (years)**	**PRNT (IU/ml)**	**PRNT (Rank)**	**ELISA (lU/ml)**	**Fold of cutoff**	**OD (450 nm)**	**SD**	**Fold of cutoff**	**OD (450 nm)**	**SD**	**Fold of cutoff**	**OD (450 nm)**	**SD**	**Fold of cutoff**	**OD (450 nm)**	**SD**	**Assay**
**28**	female	18	80.392	1.0	13000	13.8	1.173	0.007	8.1	1.051	0.035	6.0	0.520	0.074	2.8	0.464	0.063	1
**24**	female	1.7	51.667	2.0	15000	14.1	1.199	0.047	7.6	0.982	0.031	6.3	0.552	0.027	2.7	0.455	0.075	1
**27**	female	40	32.890	3.0	11000	12.1	1.031	0.235	8.0	1.034	0.025	6.0	0.514	0.016	2.9	0.485	0.071	1
**35**	male	27	11.549	4.0	9000	26.1	2.247	0.030	13.5	1.420	0.184	3.8	0.369	0.002	12.1	1.296	0.009	2
**IVIG**			11.549	5.0	9000	10.6	0.901	0.016	6.8	0.875	0.129	5.1	0.448	0.001	2.8	0.468	0.004	1
**31**	female	90	10.779	6.0	13000	20.9	1.800	0.028	12.2	1.283	0.097	2.5	0.250	0.006	14.0	1.498	0.071	2
**32**	female	47	3.909	7.0	7100	21.7	1.867	0.069	12.9	1.356	0.008	1.6	0.152	0.001	13.3	1.427	0.066	2
**18**	male	63	3.596	8.0	3000	10.3	0.876	0.133	5.8	0.744	0.104	3.3	0.287	0.033	2.5	0.417	0.051	1
**39**	female	43	3.179	9.0	9100	17.0	1.552	0.074	8.2	0.940	0.107	7.3	0.600	0.010	4.2	0.459	0.053	3
**11**	male	31	3.092	10.0	12000	12.3	1.049	0.063	8.6	1.103	0.047	6.8	0.589	0.047	2.6	0.429	0.034	1
**19**	male	27	2.382	11.0	3300	6.2	0.525	0.000	5.1	0.655	0.020	1.4	0.123	0.008	3.5	0.579	0.058	1
**40**	male	20	1.691	12.0	5600	11.8	1.077	0.076	2.3	0.268	0.011	1.6	0.133	0.011	1.9	0.194	0.007	3
**41**	female	29	1.691	13.0	1900	2.6	0.238	0.018	1.5	0.175	0.008	1.0	0.083	0.013	1.4	0.152	0.046	3
**38**	female	24	1.376	14.0	1000	13.5	1.232	0.022	2.2	0.258	0.011	2.1	0.177	0.007	2.1	0.233	0.015	3
**1**	female	28	1.090	15.0	3200	3.9	0.335	0.018	3.0	0.387	0.003	2.9	0.251	0.013	1.6	0.273	0.039	1
**36**	male	27	1.040	16.0	4800	18.9	1.565	0.168	8.6	0.803	0.056	**0.9**	0.095	0.008	8.1	0.865	0.058	2
**37**	male	17	0.728	17.0	510	7.8	0.681	0.093	1.8	0.213	0.023	1.7	0.145	0.063	1.1	0.117	0.004	3
**26**	female	27	0.654	18.0	1000	16.2	1.390	0.013	5.9	0.620	0.084	2.0	0.191	0.006	3.9	0.422	0.028	2
**17**	male	6	0.627	19.0	1000	2.7	0.233	0.015	2.9	0.371	0.011	1.1	0.088	0.004	2.2	0.366	0.068	1
**6**	male	13	0.554	20.0	1100	1.8	0.160	0.020	2.0	0.260	0.018	1.6	0.101	0.013	1.8	0.292	0.013	1

**33**	male	31	0.541	21.0	430	4.4	0.376	0.001	2.2	0.235	0.002	**0.9**	0.082	0.003	2.1	0.222	0.007	2
**2**	female	30	0.455	22.0	1000	2.6	0.160	0.006	2.7	0.348	0.014	1.3	0.117	0.007	1.9	0.309	0.010	3
**16**	female	37	0.408	23.0	3400	2.4	0.208	0.009	2.4	0.309	0.018	1.5	0.138	0.011	1.6	0.268	0.040	1
**21**	female	13	0.387	24.0	3300	10.0	0.848	0.013	6.0	0.776	0.117	5.5	0.483	0.009	2.8	0.473	0.018	1
**34**	male	24	0.358	25.0	930	8.3	0.714	0.047	3.6	0.383	0.067	**0.7**	0.064	0.011	3.0	0.326	0.011	2
**29**	female	18	0.344	26.0	370	3.1	0.266	0.012	2.9	0.303	0.099	1.1	0.108	0.010	1.6	0.167	0.030	2
**4**	female	26	0.156	27.0	270	2.8	0.237	0.047	1.5	0.158	0.023	**0.9**	0.083	0.010	2.0	0.218	0.005	2
**30**	female	23	0.097	28.0	860	1.5	0.134	0.008	1.5	0.154	0.014	**0.9**	0.084	0.003	1.5	0.160	0.010	2
**8**	female	37	0.074	29.0	400	1.6	0.139	0.011	**0.8**	0.084	0.004	1.1	0.106	0.022	**0.9**	0.100	0.008	2
**10**	female	21	0.072	30.0	240	1.3	0.114	0.018	1.2	0.127	0.006	1.0	0.099	0.001	**0.9**	0.097	0.011	2
**5**	female	6	0.052	31.0	150	0.9	0.080	0.006	0.9	0.116	0.021	0.9	0.081	0.001	0.8	0.137	0.005	1
**3**	male	16	0.030	32.0	150	0.9	0.073	0.002	0.7	0.092	0.004	0.9	0.080	0.007	0.9	0.149	0.001	1
**7**	male	21	0.001	33.0	150	**1.0**	0.095	0.001	0.9	0.095	0.001	0.9	0.089	0.011	0.8	0.082	0.019	2
**9**	male	35	0.001	34.0	150	**1.0**	0.098	0.000	0.9	0.088	0.002	0.9	0.089	0.002	0.9	0.091	0.001	2
**12**	female	24	0.001	35.0	150	0.8	0.078	0.004	**1.4**	0.166	0.011	0.7	0.055	0.002	0.8	0.089	0.006	3
**13**	female	40	0.001	36.0	150	0.8	0.077	0.001	0.8	0.084	0.017	0.8	0.077	0.005	0.8	0.075	0.002	3
**14**	male	16	0.001	37.0	150	0.5	0.048	0.001	0.4	0.048	0.001	0.6	0.047	0.001	0.5	0.045	0.001	3
**15**	male	6	0.001	38.0	150	0.6	0.053	0.004	0.5	0.052	0.004	0.7	0.054	0.004	0.6	0.053	0.004	3
**20**	male	53	0.001	39.0	150	0.8	0.080	0.027	0.7	0.079	0.027	0.9	0.081	0.027	0.8	0.080	0.027	3
**22**	female	16	0.001	40.0	150	0.6	0.056	0.010	0.5	0.056	0.010	0.7	0.057	0.010	0.6	0.060	0.010	3
**23**	female	14	0.001	41.0	150	0.7	0.065	0.012	0.6	0.065	0.012	0.8	0.066	0.012	0.6	0.052	0.012	3
**25**	female	26	0.001	42.0	150	0.7	0.060	0.001	0.5	0.061	0.001	0.8	0.059	0.001	0.7	0.063	0.001	3
**cutoff**			0.055		151#	1.0			1.0			1.0			1.0			
The totality of the samples was measured in 3 independently performed parts (1-3), each resulting in a different cut off value																		
**1**							0.085			0.129			0.087			0.157		
**2**							0.086			0.105			0.096			0.107		
**3**							0.091			0.115			0.082			0.108		
In Bold: above or below cut off value																		

Manufacturer´s cut off value

TABLE S2: Raw data of individual human sera for MV immune IgG reaction patterns. Measurements of IgG UI/ml (Enzygnost), serum dilution for 50% PRNT, fold from cut off, OD at 450 nm and standard deviations of each BW:FcγR-ζ reporter cell assay and of the different sera tested for MV-specific IgG detection are listed. Positive values were set if reaching cut off values ≥ 151 for ELISA, ≥ 1 for each BW:FcγR-ζ assay and ≥ 0.055 for the neutralization assay. Positive and negative results are separated by a bold line. Results highlighted in bold were below the value defined as positive for that particular assay in the case of the positive samples or above the value defined as negative for that particular assay in the case of the negative samples. Samples were measured in triplicates. The totality of the samples was measured in three independently performed sub experiments (1, 2, 3), resulting in slightly different cut off values as indicated.
