# Supplementary material for: Highly individual patterns of virus-immune IgG effector responses in humans
Source: Med Microbiol Immunol. 2016 May 18;205(5):409–24. doi: 10.1007/s00430-016-0457-y (PMC5003914; doi:10.1007/s00430-016-0457-y)
Supplement: Supplementary file 5 — Supplementary material 5 (PDF 241 kb) [file 430_2016_457_MOESM5_ESM.pdf]

Table S3: Raw data for MV immune IgG reaction patterns of vaccinees with low/negative MV-IgG ELISA responses.

				FcγRIIIA-ζ activation			FcγRIIA-ζ activation			FcγRIIB-ζ activation			FcγRI-ζ activation		
**Donor no.**	**PRNT (IU/ml)**	**PRNT IgG (Rank)**	**ELISA (lU/ml)**	**Fold of cutoff**	**OD (450 nm)**	**SD**	**Fold of cutoff**	**OD (450 nm)**	**SD**	**Fold of cutoff**	**OD (450 nm)**	**SD**	**Fold of cutoff**	**OD (450 nm)**	**SD**
**736**	1.183	1.0	220	2.0	0.181	0.001	1.6	0.146	0.011	1.0	0.089	0.008	1.3	0.124	0.013
**723**	0.918	2.0	100	1.7	0.152	0.001	1.4	0.131	0.007	1.0	0.096	0.001	1.1	0.098	0.012
**731**	0.860	3.0	160	3.3	0.304	0.011	2.6	0.239	0.004	1.3	0.118	0.006	1.7	0.156	0.002
**760**	0.770	4.0	320	1.8	0.161	0.011	1.7	0.159	0.036	1.1	0.098	0.004	1.1	0.103	0.011
**728**	0.648	5.0	40	1.8	0.168	0.004	1.2	0.110	0.009	1.1	0.105	0.007	1.2	0.113	0.004
**750**	0.647	6.0	250	1.3	0.124	0.003	1.2	0.107	0.004	1.1	0.099	0.004	1.2	0.109	0.015
**733**	0.627	7.0	150	2.7	0.246	0.002	1.9	0.170	0.011	1.3	0.122	0.006	1.3	0.124	0.023
**748**	0.521	8.0	210	1.7	0.156	0.006	1.2	0.114	0.001	1.2	0.111	0.011	1.2	0.108	0.008
**766**	0.439	9.0	170	1.6	0.151	0.016	1.3	0.124	0.005	1.2	0.107	0.011	1.3	0.119	0.008
**746**	0.428	10.0	100	2.0	0.179	0.011	1.6	0.149	0.009	1.3	0.122	0.006	1.4	0.127	0.006
**735**	0.394	11.0	120	1.5	0.134	0.018	1.1	0.101	0.005	1.1	0.099	0.008	1.1	0.104	0.003
**732**	0.385	12.0	260	2.7	0.243	0.004	2.5	0.229	0.006	1.4	0.131	0.000	1.3	0.117	0.009
**721**	0.342	13.0	180	3.1	0.284	0.033	2.1	0.192	0.025	1.3	0.118	0.001	1.2	0.111	0.007
**724**	0.340	14.0	30	1.8	0.164	0.022	1.3	0.120	0.008	1.1	0.099	0.002	1.4	0.127	0.002
**741**	0.319	15.0	190	1.6	0.143	0.011	1.3	0.120	0.011	1.4	0.132	0.007	1.3	0.120	0.006
**727**	0.309	16.0	0	1.6	0.147	0.016	1.4	0.126	0.035	1.2	0.115	0.023	1.2	0.114	0.012
**751**	0.241	17.0	140	1.8	0.167	0.010	1.5	0.134	0.008	1.3	0.123	0.001	2.7	0.124	0.002
**725**	1.268	18.0	210	2.7	0.244	0.029	1.7	0.156	0.012	1.2	0.108	0.008	1.8	0.169	0.005
**cutoff**	0.200			1.0	0.092		1.0	0.092		1.0	0.092		1.0	0.092	

TABLE S3: Raw data of vaccinees with low MV-IgG ELISA responses. IgG UI/ml (Enzygnost), fold from cutoff and OD at 450 nm for each BW:FcγR-ζ reporter cell assay and serum dilution for 50% PRNT of the different sera tested for specific MV IgG are listed. Borderline values for Elisa were set if ≥ 0 and ≤ 320 .Positive values were set if ≥ 1 for each BW:FcγR-ζ assay and ≥ 0.200 for neutralization. Samples were measured in triplicates.
