# Supplementary material for: Highly individual patterns of virus-immune IgG effector responses in humans
Source: Med Microbiol Immunol. 2016 May 18;205(5):409–24. doi: 10.1007/s00430-016-0457-y (PMC5003914; doi:10.1007/s00430-016-0457-y)
Supplement: Supplementary file 6 — Supplementary material 6 (PDF 312 kb) [file 430_2016_457_MOESM6_ESM.pdf]

Table S4: Raw data for HCMV immune IgG reaction patterns of individual human sera.

						FcγRIIIA-ζ activation			FcγRIIA-ζ activation			FcγRIIB-ζ activation			FcγRI-ζ activation			
**Donor no.**	**Sex**	**Age (years)**	**PRNT (1/Serum dilution)**	**PRNT (Rank)**	**ELISA (lU/ml)**	**Fold of cutoff**	**OD (450 nm)**	**SD**	**Fold of cutoff**	**OD (450 nm)**	**SD**	**Fold of cutoff**	**OD (450 nm)**	**SD**	**Fold of cutoff**	**OD (450 nm)**	**SD**	**Assay**
**44**	FEMALE	39	3584.0	1.0	12.0	6.8	0.574	0.023	2.6	0.265	0.013	1.9	0.159	0.030	1.4	0.133	0.001	2
**49**	MALE	45	2850.9	2.0	14.6	3.5	0.531	0.051	1.0	0.189	0.016	1.3	0.175	0.004	1.0	0.210	0.008	1
**29**	FEMALE	18	1160.0	3.0	22.0	6.5	0.957	0.036	1.2	0.238	0.019	2.2	0.286	0.011	1.5	0.320	0.003	1
**45**	FEMALE	66	1094.0	4.0	22.0	6.4	0.544	0.071	2.6	0.249	0.001	1.9	0.109	0.008	1.4	0.131	0.004	2
**46**	MALE	45	1057.8	5.0	22.0	3.9	0.336	0.060	1.8	0.181	0.027	1.4	0.114	0.001	1.3	0.121	0.001	2
**50**	FEMALE	30	834.5	6.0	6.2	2.4	0.352	0.034	1.6	0.262	0.007	1.9	0.246	0.057	**0.6**	0.084	0.016	1
**9**	MALE	35	789.1	7.0	16.5	3.8	0.569	0.066	**0.9**	0.178	0.006	1.2	0.161	0.008	1.0	0.213	0.018	1
**27**	FEMALE	40	734.0	8.0	22.0	3.1	0.463	0.048	**0.9**	0.192	0.002	1.8	0.243	0.050	1.0	0.218	0.008	1
**19**	MALE	27	729.3	9.0	6.9	7.0	0.598	0.072	2.1	0.207	0.016	1.7	0.137	0.030	1.6	0.156	0.005	2
**47**	MALE	69	599.1	10.0	8.2	2.2	0.186	0.014	2.1	0.207	0.021	1.5	0.122	0.012	1.3	0.126	0.017	2
**IVIG**			590.3	11.0	22.0	9.6	0.820	0.086	2.6	0.263	0.013	1.8	0.154	0.008	1.7	0.164	0.006	2

**2**	FEMALE	30	501.1	12.0	9.2	2.7	0.400	0.032	**0.7**	0.127	0.010	1.8	0.238	0.049	1.4	0.306	0.006	1
**20**	MALE	53	407.3	13.0	22.0	4.8	0.715	0.017	**0.7**	0.131	0.008	1.3	0.165	0.027	1.5	0.336	0.008	1
**12**	FEMALE	24	348.4	14.0	13.2	2.4	0.356	0.031	**0.8**	0.151	0.062	1.1	0.142	0.001	**0.6**	0.140	0.050	1
**6**	MALE	13	317.6	15.0	10.0	1.8	0.263	0.007	**0.8**	0.154	0.035	1.1	0.148	0.015	**0.4**	0.081	0.029	1
**16**	FEMALE	37	253.3	16.0	7.5	2.4	0.356	0.057	**0.8**	0.160	0.052	1.5	0.193	0.037	**0.9**	0.195	0.016	1
**48**	MALE	15	252.9	17.0	5.7	1.7	0.252	0.046	1.0	0.190	0.010	1.3	0.168	0.020	**0.4**	0.092	0.004	1
**52**	FEMALE	2	196.3	18.0	9.7	8.2	0.297	0.002	1.1	0.088	0.003	2.3	0.100	0.012	3.1	0.129	0.023	3
**42**	MALE	55	143.3	19.0	11.4	3.1	0.262	0.029	1.9	0.189	0.012	1.5	0.128	0.036	1.1	0.103	0.001	2
**17**	MALE	6	124.0	20.0	22.0	3.6	0.533	0.055	**0.9**	0.180	0.026	1.8	0.232	0.008	**0.7**	0.160	0.042	1
**15**	MALE	6	101.1	21.0	7.4	2.1	0.186	0.005	1.9	0.186	0.011	1.0	0.081	0.001	1.3	0.124	0.030	2
**25**	FEMALE	26	98.8	22.0	2.1	2.6	0.223	0.013	2.0	0.200	0.008	1.9	0.157	0.005	1.5	0.147	0.011	2
**26**	FEMALE	27	70.0	23.0	5.8	2.8	0.236	0.001	1.5	0.147	0.004	1.6	0.133	0.021	1.2	0.121	0.011	2
**43**	FEMALE	51	30.0	24.0	2.2	2.3	0.198	0.006	1.5	0.154	0.011	1.1	0.092	0.006	1.0	0.092	0.006	2
**51**	FEMALE	0.2	30.0	25.0	1.0	2.3	0.084	0.000	**0.8**	0.059	0.004	1.8	0.078	0.008	1.6	0.068	0.001	3
**22**	FEMALE	16	7.8	26.0	4.1	1.8	0.156	0.006	1.2	0.121	0.016	1.7	0.142	0.040	1.3	0.127	0.018	2
**1**	FEMALE	28	5.0	27.0	4.2	2.4	0.204	0.004	1.6	0.165	0.013	2.3	0.191	0.000	1.3	0.124	0.013	2
**53**	FEMALE	67	5.0	28.0	4.2	2.4	0.088	0.001	1.7	0.135	0.008	3.1	0.134	0.018	1.8	0.076	0.012	3
**3**	MALE	16	5.0	29.0	0.2	0.6	0.102	0.006	0.6	0.120	0.008	0.8	0.114	0.006	0.8	0.149	0.005	1

**4**	FEMALE	26	5.0	30.0	0.2	0.9	0.139	0.030	0.6	0.118	0.011	0.9	0.130	0.015	0.6	0.135	0.038	1
**5**	FEMALE	6	5.0	31.0	0.2	0.8	0.072	0.002	0.9	0.074	0.006	0.9	0.070	0.001	0.8	0.076	0.004	2
**7**	MALE	21	5.0	32.0	0.2	0.9	0.084	0.008	0.9	0.088	0.001	0.8	0.069	0.016	0.7	0.072	0.000	2
**8**	FEMALE	37	5.0	33.0	0.2	**1.0**	0.040	0.001	0.7	0.044	0.013	**1.0**	0.052	0.003	0.9	0.035	0.008	3
**10**	FEMALE	21	5.0	34.0	0.2	**1.0**	0.037	0.001	0.7	0.041	0.014	**1.1**	0.055	0.031	0.9	0.035	0.004	3
**11**	MALE	31	5.0	35.0	0.2	0.5	0.040	0.002	0.6	0.039	0.002	0.5	0.045	0.008	0.6	0.024	0.001	2
**13**	FEMALE	40	5.0	36.0	0.2	0.6	0.036	0.000	0.5	0.033	0.005	0.8	0.035	0.002	0.9	0.038	0.002	2
**14**	MALE	16	5.0	37.0	0.2	0.6	0.022	0.004	0.3	0.041	0.011	0.9	0.041	0.011	0.9	0.038	0.007	3
**18**	MALE	63	5.0	38.0	0.2	0.8	0.029	0.001	0.2	0.037	0.002	0.6	0.046	0.008	0.5	0.048	0.004	3
**21**	FEMALE	13	5.0	39.0	0.2	0.8	0.052	0.002	0.6	0.055	0.006	0.5	0.043	0.011	0.6	0.048	0.008	2
**23**	FEMALE	14	5.0	40.0	0.2	0.5	0.016	0.003	0.8	0.046	0.003	0.5	0.026	0.003	0.4	0.019	0.003	3
**24**	FEMALE	1.7	5.0	41.0	0.2	0.6	0.025	0.001	0.4	0.034	0.007	0.9	0.032	0.010	0.9	0.039	0.001	3
**28**	FEMALE	18	5.0	42.0	0.2	0.5	0.019	0.005	0.3	0.028	0.011	0.6	0.024	0.006	0.4	0.020	0.005	3
**CUTOFF**			6.0		0.3#	1.0			1.0			1.0			1.0			
The totality of the samples was measured in 3 independently performed parts (1-3), each resulting in a different cut off value																		
**1**							0.148			0.193			0.132			0.220		
**2**							0.085			0.100			0.084			0.095		

**3**							0.036			0.077			0.043			0.041		
In Bold: above or below cut off value																		
# Manufacturer´s cut off value																		

TABLE S4: Raw data of individual human sera. IgG U/ml (LIAISON), serum dilution for 50% PRNT, fold from cut off, OD at 450 nm and standard deviations for each BW:FcγR-ζ reporter cell assay and of the different sera tested for specific HCMV IgG are listed. Positive values were set if ≥ 0.3 for ELISA, ≥ 1 for each BW:FcγR-ζ assay and ≥ 6 for neutralization. Positive and negative results are separated by a bold line. Results highlighted in bold were below the value defined as positive for that particular assay in the case of the positive samples or above the value defined as negative for that particular assay in the case of the negative samples. Samples were measured in triplicates. The totality of the samples was measured in three independently performed sub experiments (1, 2, 3), resulting in different cut off values as indicated.
